# Acute Restraint Stress Evokes Anxiety-Like Behavior Mediated by Telencephalic Inactivation and GabAergic Dysfunction in Zebrafish Brains

**DOI:** 10.1038/s41598-020-62077-w

**Published:** 2020-03-26

**Authors:** Nadyme Assad, Waldo Lucas Luz, Mateus Santos-Silva, Tayana Carvalho, Suellen Moraes, Domingos Luiz Wanderley Picanço-Diniz, Carlomagno Pacheco Bahia, Evander de Jesus Oliveira Batista, Adelaide da Conceição Passos, Karen Renata Herculano Matos Oliveira, Anderson Manoel Herculano

**Affiliations:** 1 Instituto de Ciências Biológicas, Universidade Federal do Pará, Belém, Pará Brazil; 2 Universidade Federal do Oeste do Pará, Santarém, Brazil; 3 Instituto de Ciências da Saúde, Universidade Federal do Pará, Belém, Pará Brazil; 4 Núcleo de Medicina Tropical, Universidade Federal do Pará, Belém, Pará Brazil; 5 Lab. Neurofarmacologia Experimental, Instituto de Ciências Biológicas, Universidade Federal do Pará, Belém, Pará Brazil

**Keywords:** Neurochemistry, Stress and resilience

## Abstract

Acute stress is an important factor in the development of anxiety disorders. Zebrafish are an organism model widely used by studies that aim to describe the events in the brain that control stress-elicited anxiety. The goal of the current study was to evaluate the pattern of cell activation in the telencephalon of adult zebrafish and the role of the GABAergic system on the modulation of anxiety-like behavior evoked by acute restraint stress. Zebrafish that underwent acute restraint stress presented decreased expression of the c-fos protein in their telencephalon as well as a significant decrease in GABA release. The data also supports that decreased GABA levels in zebrafish brains have diminished the activation of GABAA receptors eliciting anxiety-like behavior. Taken together these findings have helped clarify a neurochemical pathway controlling anxiety-like behavior evoked by acute stress in zebrafish while also opening the possibility of new perspective opportunities to use zebrafish as an animal model to test anxyolitic drugs that target the GABAergic system.

## Introduction

Anxiety is a very common behavioral disorder associated with a traumatic experience in humans^1–4^. The use of animal models to describe brain disorders related with traumatic events represents an important tool for development of new treatments and drug discoveries. Given this perspective, describing the histological and molecular events, which coordinate the brain alterations controlling animal behavior after a stressful experience, is essential to the establishment of an efficient animal model.

*Danio rerio* (zebrafish) have become an organism model widely used for studies related to normal or altered human behavior. It is justified since this specie presents a high degree of genetic, biochemical and histological homology to humans^5–10^. Zebrafish neurophysiology and behavior are regulated by the same neurotransmitter systems that are described in mammals, including humans. However the association between zebrafish behavior and neurotransmitter release in its brain remains unclear. Most of the protocols used to evaluate the altered behavior of zebrafish use an indirect approach to describe the neurochemical events in its brain. The current study aimed to describe glutamate and GABA release in zebrafish that underwent acute restraint stress. Piato *et al*. (2011) showed that zebrafish present altered behavior and biochemical alterations when submitted to acute restrain stress. Ghisleni *et al*. (2012) demonstrated that this traumatic experience evokes activation of hypothalamus-pituitary-interrenal (HPI) axis which is the fish analogous structure of mammalian hypothalamus-pituitary-adrenal (HPA) axis. Although zebrafish represent a potential animal model for studies about anxiety disorder evoked by acute stress, it remains unclear how this aversive stimulus alters the excitatory and inhibitory neurotransmission in zebrafish brain.

Glutamate and GABA represent the main excitatory and inhibitory neurotransmitters in the central nervous system, respectively. Glutamate mediates some neuropathologies by hyper stimulation of NMDA glutamate receptors and generation of neuronal excitotoxicity^11–13^. As previously reported, glutamate spillover induces neuronal loss and synaptic dysconnectivity by reduction of synaptic density. Some studies have associated these entire phenomenons with generation of anxiety disorder in humans and anxiety-like behavior in rodents^14–16^. The GABA neurotransmitter is also implicated in generation of symptoms related with anxiety disorder and anxiety-like behavior^17–19^. GABAergic signalization is mediated by stimulation of GABAA receptors which are ligand-gated ion channels and GABAB receptors which are G protein-coupled receptors^20,21^. The characterization of the roles that glutamate and GABA play in the generation of anxiety-like behavior elicited by acute stress in zebrafish is a very important step to consolidate this specie as an animal model for studies of anxiety-like behavior evoked by acute stress. Based on all these findings, the current study aimed to evaluate the pattern of brain activation of glutamate and GABA release in the brains of zebrafish that underwent acute restraint stress as well as to verify participation of glutamatergic and GABAergic systems in the anxiety-like behavior evoked by this aversive stimulus.

## Results

### Brain activation in zebrafish submitted to ARS

**Figure 1:**
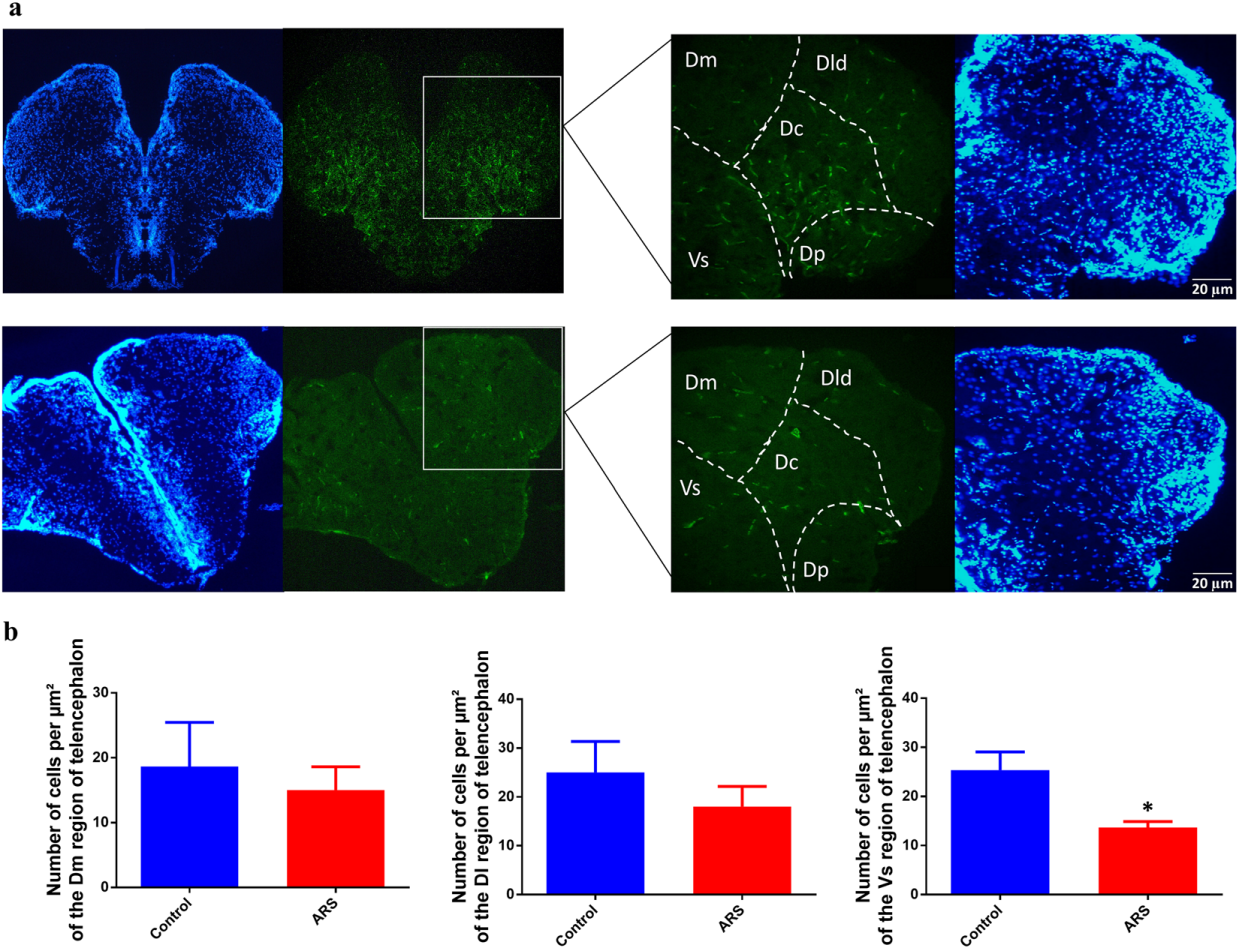
Cell activation in zebrafish brain submitted to ARS. (**a**) Imunofluorescence of dorsal telencephalon region of zebrafish brain. Top images shows DAPI staining (blue) and c-Fos staining (green) of control group and bottom images represents ARS group. Dm: dorsomedial telencephalon; Dl: dorsolateral telencephalon. Objective: 20x. (**b**) Counting of the cells marked with c-Fos in the telencephalic region of zebrafish brain. Values showed as MEAN ± S.E.M. (Student’s T test. *p < 0,05).

Firstly, we aimed to evaluate cellular activation in the brain of zebrafish submitted to ARS protocol. Our data has shown that ARS evoked a decrease in c-fos expression in zebrafish telencephalon when compared with control (Fig. 1a). This phenomenon is better demonstrated in Fig. 1b which shows the number of c-fos positive cells in telencephalic region of both groups (df = 6, p(bilateral) = 0.0002 control = 600 ± 100 cells/µm^2^ vs ARS = 250 ± 20 cells/µm^2^).

### Glutamate and GABA quantification in the brain of zebrafish submitted to ARS

**Figure 2:**
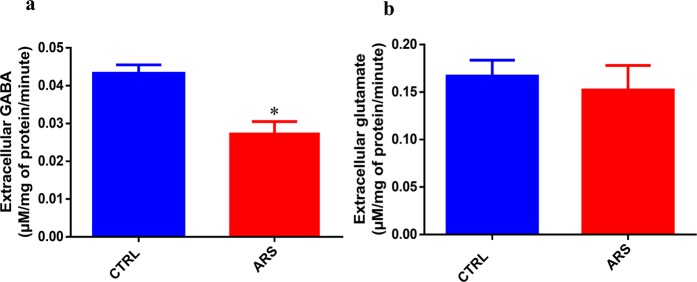
Extracellular levels of GABA (**a**) and glutamate (**b**) in the brain of zebrafish submitted to ARS. Values showed as MEAN ± S.E.M. (Student’s T test. *p < 0,05).

In the second experiment, we tried to evaluate if the ARS protocol alters neurotransmitter extracellular levels in the zebrafish brain. Glutamate and GABA were simultaneously measured in the brain samples and, as demonstrated in Fig. 2a, ARS evokes a significant decrease in GABA extracellular levels in the zebrafish brain in comparison with control (df = 4, p(bilateral) = 0.0225 control = 0.045 ± 0.002 µM/mg ptn/minute vs ARS = 0.028 ± 0.002 µM/mg ptn/minute). Data regarding the measurements of glutamate levels (Fig. 2b) have shown that ARS did not alter glutamate extracellular levels in zebrafish brain (df = 4, p(bilateral) = 0.6582 control = 0.16 ± 0.02 µM/mg ptn/minute vs ARS = 0.15 ± 0.04 µM/mg ptn/minute).

### Pharmacological evaluation of anxiety-like behavior induced by ARS

**Figure 3:**
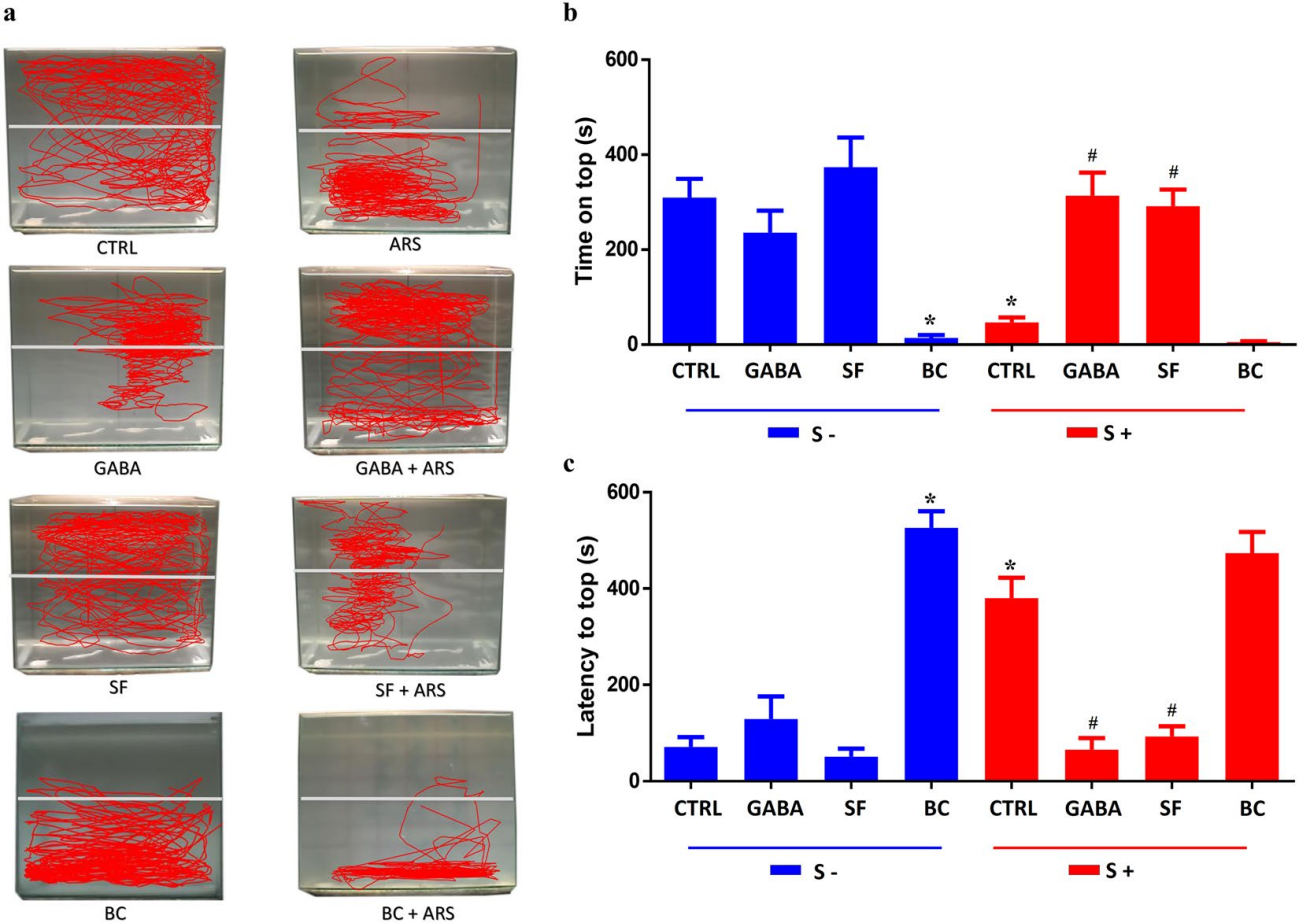
Effects of GABA, bicuculline and saclofen in locomotion (**a**) and behavior parameters of zebrafish: time spent on top in seconds (**b**) and latency to top in seconds. (**c**) Animals that did not went through the ARS are represented as S- and those who were submitted to ARS are represented as S+. Values showed as MEAN ± S.E.M. (ANOVA one way, Tukey post test. *Compared to control (S−) #compared to control (S+) p < 0,05).

Since our results demonstrated that ARS altered only GABA release, we tried to evaluate if this neurochemical alteration could be related to behavioral changes induced by ARS. In the novel tank test, time on the top (Fig. 3a) showed that the ARS group spent less time on the top of the apparatus than control group (F_(1, 81)_ = 9.21, df = 1, p < 0.05 control = 309.9 ± 37.4 s vs ARS = 46.9 ± 9.6 s). This anxiety-like behavior was also evidenced when we evaluated latency to top. As demonstrated in Fig. 3b, animals submitted to ARS took a longer time to go to top of apparatus (F_(1, 83)_ = 6.13, df = 1, p < 0.01 control = 71 ± 19.8 s vs ARS = 380 ± 41 s). Our data also showed that the anxiogenic effect elicited by ARS was prevented by GABA treatment. This phenomenon was observed in two parameters: time on top (F_(3, 81)_ = 38.6, df = 3, p < 0.05 control = 309 ± 37.4 s vs ARS = 46.9 ± 9.6 s vs GABA + ARS = 313.5 ± 43.6 s) and latency to top (F_(3, 83)_ = 68.2, df = 3, p < 0.01 control = 71 ± 19.8 s vs ARS = 380 ± 41 s vs GABA + ARS = 65.9 ± 22.6 s). Data regarding squares crossed and freezing evaluation were not altered by ARS which suggests that acute stress did not alter zebrafish motility or exert panicogenic-like behavior.

**Figure 4:**
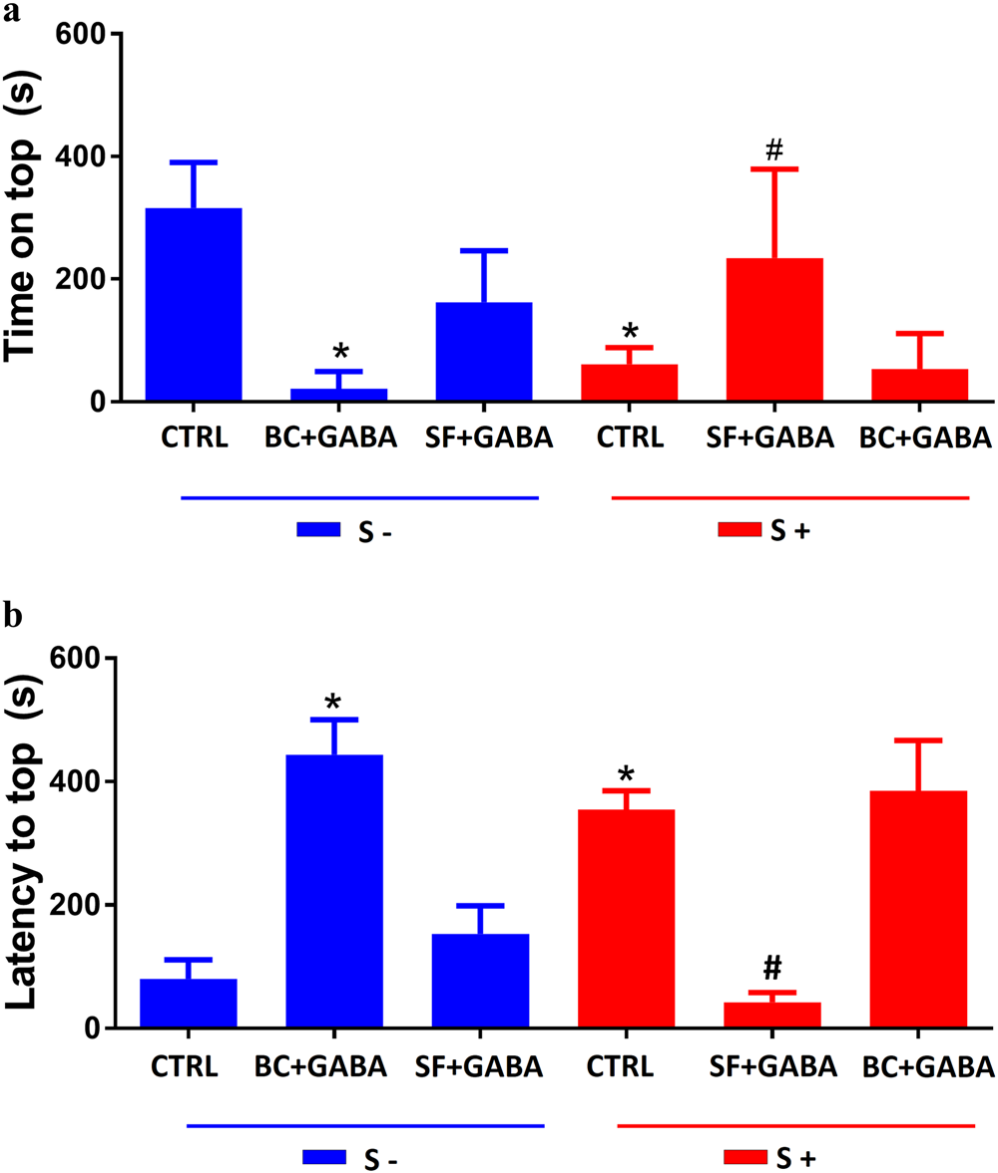
Effects of co-treatment of GABA, bicuculline and saclofen in behavior parameters of zebrafish: time spent on top in seconds (**a**) and latency to top in seconds (**b**). Animals that did not went through the ARS are represented as S- and those who were submitted to ARS are represented as S+. Values showed as MEAN ± S.E.M. (ANOVA one way, Tukey post test. *Compared to control (S−) ^#^compared to control (S+) p < 0,05).

The preventive effect of GABA on anxiety-like behavior elicited by ARS leads us to ask which GABAergic receptors could be involved in this phenomenon. Our data has shown that treatment with bicuculline, an antagonist of GABAA receptors, inhibited the GABA preventive effect on anxiety-like behavior evoked by ARS. This phenomenon was observed in both time on top (F_(2, 27)_ = 24.8, df = 2, p < 0.05 controlλ = 315 ± 32 s vs ARS = 54.4 ± 8.1 s vs GABA = 373.8 ± 58.5 s vs BC + GABA + ARS λ = 21.4 ± 9.7 s) and latency to top (F_(2, 24)_ = 24.18, df = 2, p < 0.01 control = 50.5 ± 9.1 s vs ARS = 380.3 ± 35.3 s vs GABA = 129.2 ± 45 s vs BC + GABA + ARS = 385 ± 73.1 s) as shown in Fig. 4. On the other hand, treatment with saclofen, a recognized antagonist of GABAB receptors, had no effect on the anxiolytic effect of GABA in ARS group (Fig. 4). The role of GABAA receptors as controlling agent of anxiety-like behavior in zebrafish was also observed in non-stressed animals. As observed in Fig. 3, bucuculline evoked significant anxiogenic-like in these subjects. However, no significant behavioral alterations were observed in non-stressed animals which were treated with saclofen (Fig. 3).

## Discussion

The current study shows for the first time that acute restraint stress induces inactivation of telencephalic region and decrease of GABA release in zebrafish brains. These finds give significant contributions for literature since zebrafish represent an important organism model for studies regard behavioral disorders induced by acute stress.

Acute restrain stress is a potent inductor of posttraumatic behavior disorders such as anxiety and panic syndrome. In mammalians, this aversive stimulus affects limbic system triggering behavioral impairments in response to stress^22,23^. In fact, previous studies have already shown that amygdale represents a limbic structure involved in generation of anxiety-like behavior evoked by acute stress. In the current study we have shown that acute restraint stress evokes substantial inactivation of telencephalic region of zebrafish brain which is described as amygdale-like region of teleost^24,25^. Morphofunctional studies also have described that amygdale-like region in teleost is housed between ventral and dorsal region of zebrafish telencephalon. Some studies support that dorsal region of teleost telencephalon (Dm) is homologous to pallial amygdala while their ventral regions (Vd, Vs and Vp) are homologous of the subpallial amygdala^26–28^. In our histological slices was not possible to evaluate the ypsiloniform sulcus (Vp subregion of zebrafish telencephalon). However, we have shown that acute restrain stress evoked significant decrease inactivation of Vs region, but not in Dm and DI dorsal subregions. It was previously described that Dm region presents intense number of glutamatergic neurons while in the Vs region there is a predominance of GABAergic neurons. Taken together these finds support our neurochemical results which have shown a predominant effect of ARS on the GABAergic system when compared with glutamatergic system in zebrafish brain.

**Figure 5:**
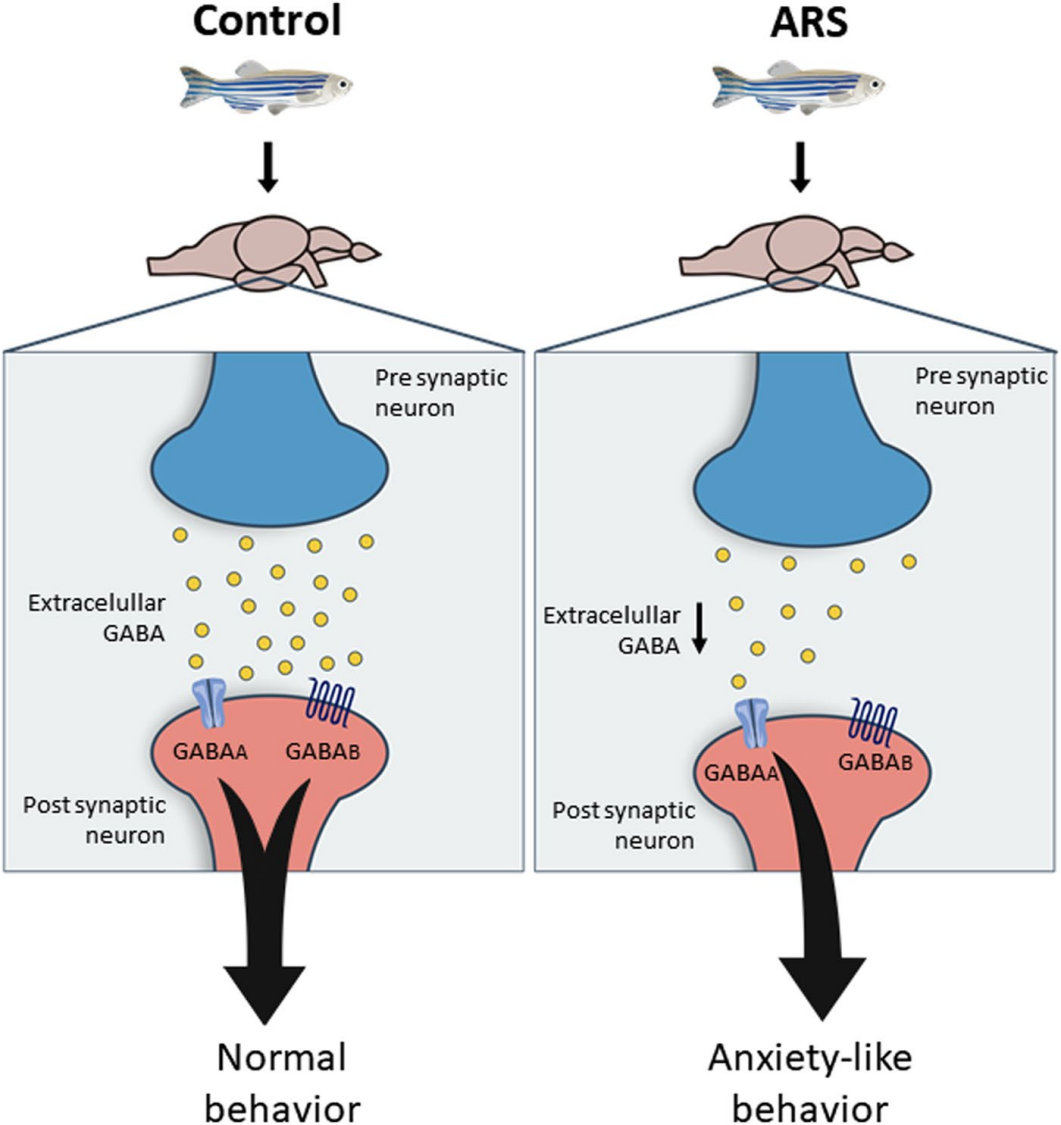
Representative image of GABAergic signaling in zebrafish brain under normal and ARS conditions.

Data presented in current study show that acute restraint stress alters GABAergic system by decrease of GABA release in the zebrafish brain. This effect was not observed when glutamate release was evaluated in animals submitted to acute restraint stress. The evidences that there are no alterations in extracellular glutamate levels associated with acute restraint stress suggest that the behavioral disorder described was mainly mediated by GABAergic disruption in zebrafish brain. Our data let us to support the hypothesis that acute restraint stress induces anxiety-like behavior in zebrafish by decrease of GABA release leading to low activation of GABAA receptors. In other words, GABA seems to assure the normal pattern of anxiety behavior by constant and tonic stimulation of GABAA receptors. This hypothesis is ratified by our results showing that GABA treatment prevented anxiety-like behavior evoked by acute restraint stress. In addition, inhibition of GABAA receptors blocked preventive action of GABA, being this effect not observed in animals treated with GABAB antagonist. The participation of GABAergic system as a “protagonist” in the generation of anxiety disorder is well documented in mammalian animal models as well as in humans^29–32^ and the current study demonstrated that zebrafish possess similar pattern of GABAergic response and brain activation. As presented in Fig. 5, the current study has lighted important neurochemical alterations associated with anxiety-like behavior evoked by acute restraint stress in zebrafish. In addition, our results open new perspectives for use of zebrafish as organism model for test of drugs able to modulate GABAergic system and behavior disorders induced by acute stress.

## Methods

### Drugs and reagents

Drugs used in the current study were: GABA (γ-Aminobutyric acid; CAS number: 56-12-2, GABAa and GABAb receptor agonists), Bicuculline ((6*R*)-6-[(5*S*)-6-methyl-5,6,7,8-tetrahydro[1,3]dioxolo[4,5-*g*]isoquinolin-5-yl]furo[3,4-*e*][1,3]benzodioxol-8(6*H*)-one; CAS number: 485-49-4, selective GABAa receptor antagonist) and Saclofen (β-(Aminomethyl)-4-chlorobenzeneethanesulfonic acid; CAS Number: 125464-42-8, selective GABAb receptor antagonist).

### Animals and housing

144 adult *Danio rerio* (zebrafish) longfin *wildtype* from both sexes (50:50 ratio), n = 6–8 per group, were purchased from a local distributor (Ananindeua – PA). The animals were kept under adequate acclimation conditions for at least one week before the experiments. Fish were kept in a light/dark cycle (14 h/10 h), fed twice a day and housed in 50 L tanks (50 × 35 × 30) at 25 °C ± 2 °C, pH = 6.5, with density of 1 animal per liter. All tanks were maintained under constant mechanical, biological and chemical filtration. This study was previously approved by the Committee of Ethics in Research with Experimental Animals of the Federal University of Pará (CEPAE - UFPA: 213-14). All methods were performed in accordance with the guidelines of National Council of Animal Experimentation Control (CONCEA).

### Treatment and acute restraint stress protocol

All animals were randomly divided into the following groups: control, 1 mg/kg GABA, 0.5 mg/kg bicuculline, 0.5 mg/kg saclofen, acute restraint stress (ARS), GABA + ARS, bicuculline + ARS and saclofen + ARS. All animals were anesthetized on ice (cryoanesthesia) and were pre-treated by intraperitoneal (i.p.) injections using *Hamilton®* syringe. Control and ARS groups only received saline injections. Acute restraint stress protocol was performed as described by Piato *et al*. (2011). Briefly, after drug treatment, control and treated groups were placed inside of plastic microtubes (2 ml), with small openings at both sides, which were placed in a tank for 90 minutes. The control, GABA, bicuculline and saclofen groups were kept under similar experimental conditions but without any type of restraint. Animals submitted to ARS were immediately transferred to the behavioral apparatus after stress.

### C-fos immunofluorescence

Zebrafish were cryoanesthetized and quickly decapitated as previously described by Maximino *et al*. (2011). The skin and skull bones were then removed to access the brain. The tissues were fixed with 4% paraformaldehyde (PFA) solution for 2 hours and cryoprotected with crescent solution of sucrose (10%, 20% and 30%) overnight and frozen in Tissue Tek solution. This step was followed by sequential tissue sections in cryostat at 20 µm thickness. Microscope slide containing tissue sections were washed 3 times with PBS and then incubated with blocking solution (rabbit serum 5% and Triton X-100 2.5% in 0.1 M PBS) for 2 hours as described by von Trotha *et al*. (2014). Tissue sections were incubated with primary anti-c-Fos antibody (sc-52G, goat polyclonal IgG, Santa Cruz Biotechnology, 1: 400 in of Triton X-100 0.25%, rabbit serum 5%) overnight at 4 °C and then incubated with the secondary antibody (sc-3919, rabbit anti-goat IgG-TR, Texas Red conjugated; 1: 1000 in Triton X-100 solution 0.25%, rabbit serum 5%, Santa Cruz Biotechnology) for 2 hours at room temperature. One of the tissue sections didn’t receive the primary antibody being a negative control. Cell nuclei were stained with DAPI (4,6-diamine-2-fenilindol) probe and the images were visualized by fluorescence microscopy (NIKON-Eclipse). The images were recorded and the cell number in the zebrafish telencephalon was determinated using ImageJ software. Brightness and contrast were raised in the Software to help the cell couting.

### High performance liquid chromatography (HPLC)

HPLC system used in the present study was based in previous studies^33–36^ using a Shimadzu (LC-10 AD) model with an injection circuit accomplished to LC-20AT pump, analytical Shimadzu C18 column and fluorescence detector (RF-10AXL). The mobile phase (A phase) was composed by 50 mM sodium acetate, 5% methanol and 2-propanol. B phase was composed of 70% methanol. Immediately after the behavior task each animal was cryoanesthetized and quickly decapitated. After that, the skin and skull bones were removed to access the brain. Each sample was transferred to culture dishes containing 500 μl of Na^+^ -HANK buffer and maintained in a CO_2_ stove for 20 minutes at 37 °C. Analysis matrix was prepared by addition of 1% TCA followed by centrifugation for 10 minutes at 5000 rpm. Derivatization process of the analysis matrix was performed by mixing 60 μl samples in 10 μl of the methanolic o-phthaldialdehyde borate buffer pH 9.5. Final volume of samples was vortexed and injected into the system for analysis after 5 minutes. Homoserin was used as an internal standard in HPLC. Extracellular levels of glutamate and GABA were simultaneously measured in the samples and the values expressed µM/mg of protein/minute. Protein content was determined by Bradford method^37^.

### Novel tank diving test

The apparatus used in this study consists of a tank (15 × 30 × 22) completely filled with water. After treatments, each animal was individually transferred to the test apparatus and allowed to explore for 10 minutes being recorded by a camera. Time on top (s), latency to top (s), number of crossings, freezing and erratic swimming were the behavioral parameters evaluated in this task. X-Plo-Rat 2005 and ZebTrack softwares were used for the video analysis of behavior by blind experimenters^38,39^.

### Data analysis

Normal distribution of the data was evaluated by Shapiro-Wilk normality test. For the statistical analyzes comparing two groups, the data obtained was analyzed through the Student’s T test, whereas in the data referring to more than two groups, the analysis of variance (ANOVA) of two way was performed, followed by Bonferroni post test. All the statistics were made in BioEstat 5.0 software, considering statistical difference when p < 0.05.

## References

[1] Viana AG (2017). Emotional clarity, anxiety sensitivity, and PTSD symptoms among trauma-exposed inpatient adolescents. Child. Psychiatry Hum. Dev..

[2] Hovens JG, Giltay EJ, Spinhoven P, van Hemert AM, Penninx BW (2015). Impact of childhood life events and childhood trauma on the onset and recurrence of depressive and anxiety disorders. J. Clin. Psychiatry..

[3] Price M, Van Stolk-cooke K (2015). Examination of the interrelations between the factors of PTSD, major depression, and generalized anxiety disorder in a heterogeneous trauma-exposed sample using DSM 5 criteria. J. Affect. Disord..

[4] Larsson MR, Bäckström M, Johanson A (2008). The interaction between baseline trait anxiety and trauma exposure as predictor of post-trauma symptoms of anxiety and insomnia. Scand. J. Psychol..

[5] Fontana BD, Mezzomo NJ, Kalueff AV, Rosemberg DB (2018). The developing utility of zebrafish models of neurological and neuropsychiatric disorders: A critical review. Exp. Neurol..

[6] Stewart AM, Braubach O, Spitsbergen J, Gerlai R, Kalueff AV (2014). Zebrafish models for translational neuroscience research: from tank to bedside. Trends Neurosci..

[7] Herget U, Wolf A, Wullimann MF, Ryu S (2014). Molecular neuroanatomy and chemoarchitecture of the neurosecretory preoptic-hypothalamic area in zebrafish larvae. J. Comp. Neurol..

[8] Maximino C, da Silva AWB, Gouveia A, Herculano AM (2011). Pharmacological analysis of zebrafish (Danio rerio) scototaxis. Prog. Neuropsychopharmacol. Biol. Psychiatry..

[9] Mueller T, Wullimann MF (2009). An evolutionary interpretation of teleostean forebrain anatomy. Brain Behav. Evolut..

[10] Barros TP, Alderton WK, Reynolds HM, Roach AG, Berghmans S (2008). Zebrafish: an emerging technology for *in vivo* pharmacological assessment to identify potential safety liabilities in early drug discovery. Br. J. Pharmacol..

[11] Vishnoi S, Raisuddin S, Parvez S (2016). Glutamate excitotoxicity and oxidative stress in epilepsy: modulatory role of melatonin. J. Env. Pathol. Toxicol. Oncol..

[12] Blasco H, Mavel S, Corcia P, Gordon PH (2014). The glutamate hypothesis in ALS: pathophysiology and drug development. Curr. Med. Chem..

[13] Lau A, Tymianski M (2010). Glutamate receptors, neurotoxicity and neurodegeneration. Pflug. Arch..

[14] Onaolapo OJ, Aremu OS, Onaolapo AY (2017). Monosodium glutamate-associated alterations in open field, anxiety-related and conditioned place preference behaviours in mice. Naunyn Schmiedebergs Arch. Pharmacol..

[15] Averill LA (2017). Glutamate dysregulation and glutamatergic therapeutics for PTSD: evidence from human studies. Neurosci. Lett..

[16] Rosa SG, Quines CB, Stangherlin EC, Nogueira CW (2016). Diphenyl diselenide ameliorates monosodium glutamate induced anxiety-like behavior in rats by modulating hippocampal BDNF-Akt pathway and uptake of GABA and serotonin neurotransmitters. Physiol. Behav..

[17] Mann JJ (2014). Anxiety in major depression and cerebrospinal fluid free gamma-aminobutyric acid. Depress. Anxiety..

[18] Möhler H (2012). The GABA system in anxiety and depression and its therapeutic potential. Neuropharmacology..

[19] Kalueff AV, Nutt DJ (2007). Role of GABA in anxiety and depression. Depress. Anxiety..

[20] Jembrek MJ, Vlainic J (2015). GABA Receptors: Pharmacological Potential and Pitfalls. Curr. Pharm. Des..

[21] Chebib M, Johnston GA (1999). The ‘ABC’ of GABA receptors: a brief review. Clin. Exp. Pharmacol. Physiol..

[22] Belda X, Fuentes S, Daviu N, Nadal R, Armario A (2015). Stress-induced sensitization: the hypothalamic-pituitary-adrenal axis and beyond. Stress..

[23] Herman JP, Ostrander MM, Mueller NK, Figueiredo H (2005). Limbic system mechanisms of stress regulation: hypothalamo-pituitary-adrenocortical axis. Prog. Neuropsychopharmacol. Biol. Psychiatry..

[24] Panula P (2010). The comparative neuroanatomy and neurochemistry of zebrafish CNS systems of relevance to human neuropsychiatric diseases. Neurobiol. Disease..

[25] Salas C (2006). Neuropsychology of learning and memory in teleost fish. Zebrafish..

[26] Giacomini ACVV (2016). Fluoxetine and diazepam acutely modulate stress induced-behavior. Behav. Brain Res..

[27] Von Trotha JW, Vernier P, Bally-Cuif L (2014). Emotions and motivated behavior converge on an amygdala-like structure in the zebrafish. Eur. Jour. Neuroscience..

[28] Perathoner S, Cordero-Maldonado ML, Crawford AD (2016). Potential of Zebrafish as a Model for Exploring the Role of the Amygdala in Emotional Memory and Motivational Behavior. J. Neurosci. Res..

[29] Vaiva G (2004). Low posttrauma GABA plasma levels as a predictive factor in the development of acute posttraumatic stress disorder. Biol. Psychiatry..

[30] Nemeroff CB (2003). The role of GABA in the pathophysiology and treatment of anxiety disorders. Psychopharmacol. Bull..

[31] Lydiard RB (2003). The role of GABA in anxiety disorders. J. Clin. Psychiatry..

[32] Nutt DJ (2001). Neurobiological mechanisms in generalized anxiety disorder. J. Clin. Psychiatry..

[33] Maximino C (2011). Possible role of serotoninergic system in the neurobehavioral impairment induced by acute methylmercury exposure in zebrafish (Danio rerio). Neurotoxicology Teratology.

[34] Moraes ER (2012). Determination of glutamate uptake by high performance liquid chromatography (HPLC) in preparations of retinal tissue. J. Chromatogr. B.

[35] Braga DV (2019). Adenosine A1 receptors modulate the Na+-Hypertonicity induced glutamate release in hypothalamic glial cells. Neurochemistry Int..

[36] Teixeira FB (2019). Neurochemical dysfunction in motor cortex and hippocampus impairs the behavioral performance of rats chronically exposed to inorganic mercury. J. Trace Elem. Med. Biol..

[37] Bradford MM (1976). A rapid and sensitive method for the quantitation of microgram quantittes of protein utilizing the principle of protein-dye binding. Anal. Biochem..

[38] Piato AL (2011). Acute restraint stress in zebrafish: behavioral parameters and purinergic signaling. Neurochem. Res..

[39] Ghisleni G (2012). The role of CRH in behavioral responses to acute restraint stress in zebrafish. Prog. Neuropsychopharmacol. Biol. Psychiatry..

